# Severe Heat Shock Induces Nucleolar Accumulation of mRNAs in *Trypanosoma cruzi*

**DOI:** 10.1371/journal.pone.0043715

**Published:** 2012-08-27

**Authors:** Ezequiel Názer, Ramiro E. Verdún, Daniel O. Sánchez

**Affiliations:** 1 Instituto de Investigaciones Biotecnológicas - UNSAM-CONICET, San Martín, Provincia de Buenos Aires, Argentina; 2 Leonard M. Miller School of Medicine, University of Miami, Miami, Florida, United States of America; Federal University of São Paulo, Brazil

## Abstract

Several lines of evidence have shown that, besides its traditional function in ribosome biogenesis, the nucleolus is also involved in regulating other cellular processes such as mRNA metabolism, and that it also plays an important role as a sensor and coordinator of the stress response. We have recently shown that a subset of RNA Binding Proteins and the poly(A)+ RNA are accumulated into the *Trypanosoma cruzi* nucleolus after inducing transcription inhibition with Actinomycin D. In this study, we investigated the behaviour of the *T. cruzi* mRNA population in parasites subjected to severe heat shock, an environmental stress that also decreases the rate of RNA synthesis. We found that the bulk of poly(A)+ RNA is reversibly accumulated into the nucleolus when exposing *T. cruzi* epimastigote forms to severe heat shock. However, the Hsp70 mRNA was able to bypass such nucleolar accumulation. Together, these data reinforce the idea about the involvement of the *T. cruzi* nucleolus in mRNA metabolism during an environmental stress response. Interestingly, *T. brucei* procyclic forms did not induce nucleolar accumulation of poly(A)+ RNA under such stress condition, suggesting that different trypanosomatids have adopted different responses to deal with the same stress conditions.

## Introduction

*Trypanosoma cruzi* is the etiological agent of Chagas’ disease, which affects between 16 and 18 million people, primarily in Central and South America [1]. This single-cell protozoan has a complex life cycle, alternating between an insect vector (triatomine) and mammalian hosts. As a consequence, *T. cruzi* is continuously exposed to drastic environmental changes. Therefore, in order to achieve a rapid adaptation to such conditions, *T. cruzi* requires a flexible modulation of its gene expression profile [2].

Gene expression in trypanosomes presents several differences compared to most part of the eukaryotic lineage. Among some of these differences, there is a general absence of defined promoters for protein-encoding genes [3]. In addition, transcription of mRNAs is polycistronic and constitutively generates pre-mRNAs, which are finally processed into individual mRNAs by trans-splicing and polyadenylation [2]. Trans-splicing consists in the addition of a 39-nucleotide capped RNA (known as mini-exon or Spliced Leader RNA) at the 5′end of all mRNAs [4]. Hence, it is considered that gene expression is primarily modulated at the post-transcriptional level, mainly by mRNA stability and translation control [2].

In recent years, Stress Granules (SG) and P-bodies have also emerged as another post-transcriptional layer of gene expression regulation [5]. These cytoplasmic structures have been postulated to modulate translation, operating either as protective or degradative mRNA reservoirs, especially when cells are subjected to stress [6]. In this regard, it has been shown that trypanosomes exposed to certain stress conditions, such as starvation and severe heat shock, are also able to display those cytoplasmic structures [7]–[9].

Recently, the resolution of the nucleolar proteomes of yeast, *Arabidopsis,* and humans has surprisingly shown the presence of several RNA Binding Proteins (RBPs) involved in different steps of mRNA metabolism [10], [11]. In addition, it has been reported that the nucleolus could also be involved in the regulation of other mechanisms related to mRNA metabolism, such as production of small interfering RNAs [12], maturation of microRNAs [13], accumulation of aberrantly spliced mRNAs [14], and export of viral mRNAs to the cytoplasm [15]–[17]. These observations support the notion that, besides its traditional function in ribosome biogenesis, the nucleolus could also be involved in the regulation of other cellular processes including mRNA metabolism. There is also a growing body of evidence arguing in favour of its possible role as a sensor and coordinator of the stress response [18]–[20], for instance, by sequestering key factors essential for the regulation of gene expression [10], [21], [22]. In this regard, previous works in yeast have shown that poly(A)+ RNA could be accumulated into the nucleolus in response to severe heat shock, supporting the idea that the nucleolus is involved in mRNA transport [23], [24]. Furthermore, it has been shown that whereas mRNAs transcribed from intron-containing genes are exported to the cytoplasm through a nucleolar phase, mRNAs generated from intron-less genes are not, suggesting that spliced and unspliced transcripts are exported by different pathways in fission yeast [25]. More recently, we have reported that the *T. cruzi* nucleolus is probably involved in the stress response since several RBPs and the poly(A)+ RNA are relocalized into this structure in response to transcription inhibition by Actinomycin D (ActD) [26], [27].

In this work, we found that most mRNA population is partially and reversibly accumulated into the *T. cruzi* nucleolus under an environmental stress, such as is a severe heat shock. Interestingly, the Heat shock protein70 (Hsp70) mRNA was able to bypass such nucleolar accumulation. These data reinforce the notion about the potential role of the nucleolus in the mRNA metabolism in *T. cruzi*.

## Results

### Severe Heat Shock Induces Nucleolar Accumulation of mRNAs in *T. cruzi*

**Figure 1:**
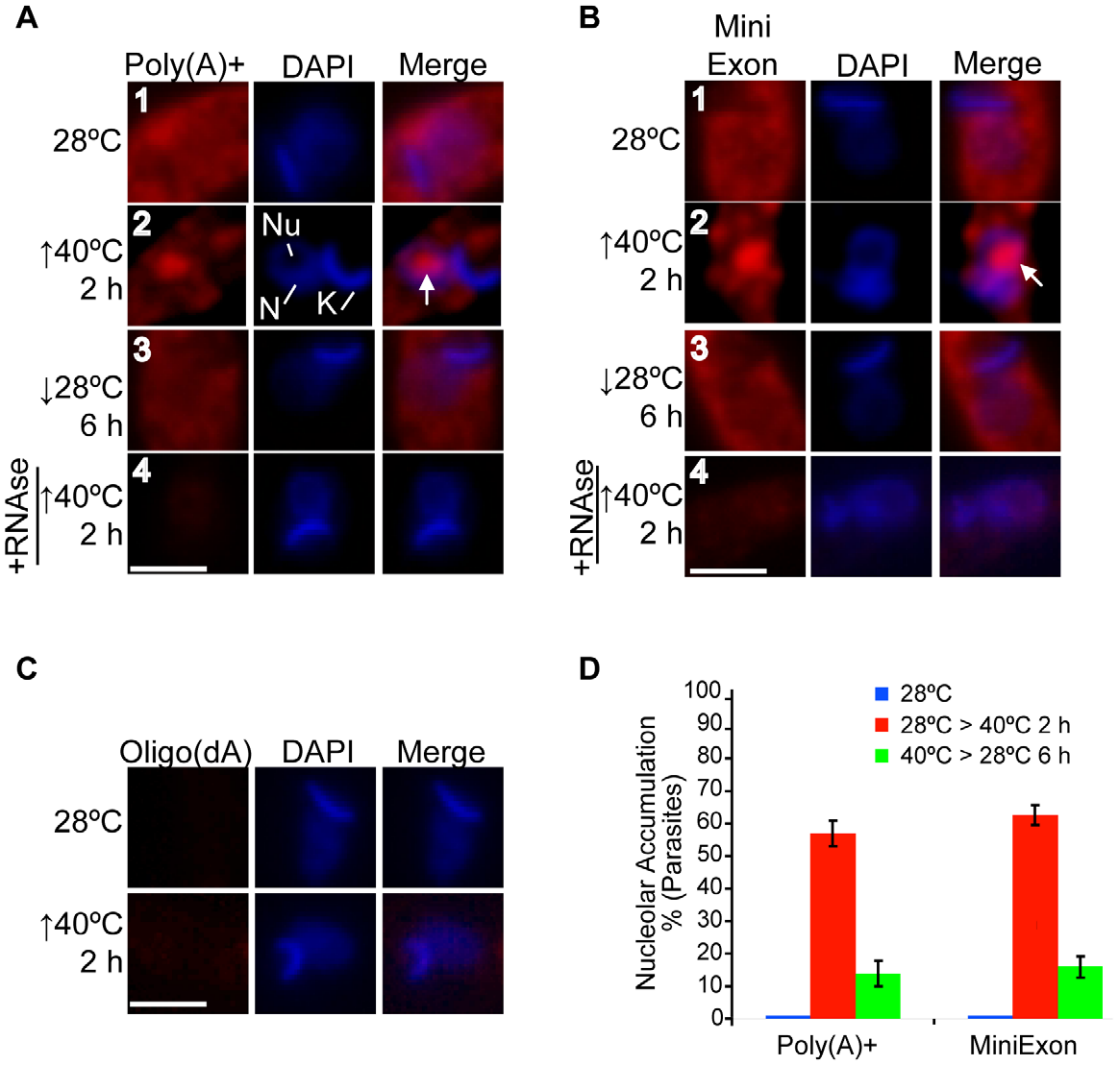
The *T. cruzi* mRNA population is partially accumulated into the nucleolus in response to severe heat shock. Localization of the mRNA population using either an (A) oligo(dT) or (B) mini-exon probe in untreated, heat-shocked and recovered *T. cruzi* epimastigotes. In addition, cells were pre-treated with RNase A before performing FISH (bottom panels). Nuclei were counterstained with DAPI (blue). The white arrows indicate the nucleolar localization for both probes. (C) RNA FISH using a Cy3-labelled oligo(dA)30 probe in untreated parasites and parasites exposed to 40°C for 2 h. N: nucleus, K: kinetoplast, Nu: nucleolus. Size bars represent 2 µm. Representative nuclei are shown. (D) A quantitative analysis of experiments is shown in panels (A) and (B). The results are expressed as mean +/− SD from at least three independent experiments (a minimum of 100 parasites per experiment were counted).

We have recently reported that poly(A)+ RNA is accumulated into the nucleolus under ActD treatment in *T. cruzi* [26]. Taken into account our previous results showing that severe heat shock stress induces the relocalization of some RBPs to the nucleolus [26], we wondered whether this stress could also induce a similar poly(A)+ RNA behaviour. To test this, we performed RNA-FISH using a Cy3-oligo(dT) probe against the poly(A) tail of mRNAs. Under normal conditions (28°C), the bulk of the poly(A)+ RNA population was distributed mainly throughout the cytoplasm, and also in the nucleus but to a much lesser extent level (Figure 1A, panel 1, Figures S1 and S3). However, when an epimastigote culture was subjected to heat shock at 40°C for 2 h, in addition to the cytoplasmic signal, a significant nucleolar accumulation of poly(A)+ RNA was observed in 57% of parasites (Figure 1A panel 2, Figures S1 and S3). To further support this result, we repeated the RNA-FISH assays using a probe against the mini-exon sequence, which is present at the 5′end of all mature mRNAs in trypanosomes. With this probe we obtained similar results, with 62% of the parasites displaying mRNA nucleolar accumulation in response to heat shock (Figure 1B, panels 1 and 2, Figures S1 and S3). As controls, we performed the following assays: i) RNase A digestion before the hybridization step (Figure 1A and B, panels 4), and ii) RNA-FISH using a Cy3-oligo(dA) probe (Figure 1C). In both control experiments, the fluorescence remaining after the hybridizations was negligible, thus confirming the probe specificity and the RNA nature of the nucleolar signals.

To find out whether the mRNA nucleolar accumulation observed was a reversible process, parasites were heat-shocked for 2 h and then re-incubated at the normal growth temperature. After 6 h at 28°C, the parasites showed an mRNA distribution similar to that of control parasites in almost the whole population (Figure 1A and B, panels 3; see Figure 1D for a quantitative analysis).

From these results, we conclude that severe heat shock in *T. cruzi* epimastigotes induces a partial relocalization of the bulk of mRNA to the nucleolus in a reversible way.

### Nucleolar Accumulation of mRNAs could be Bypassed by Hsp70 mRNAs

**Figure 2:**
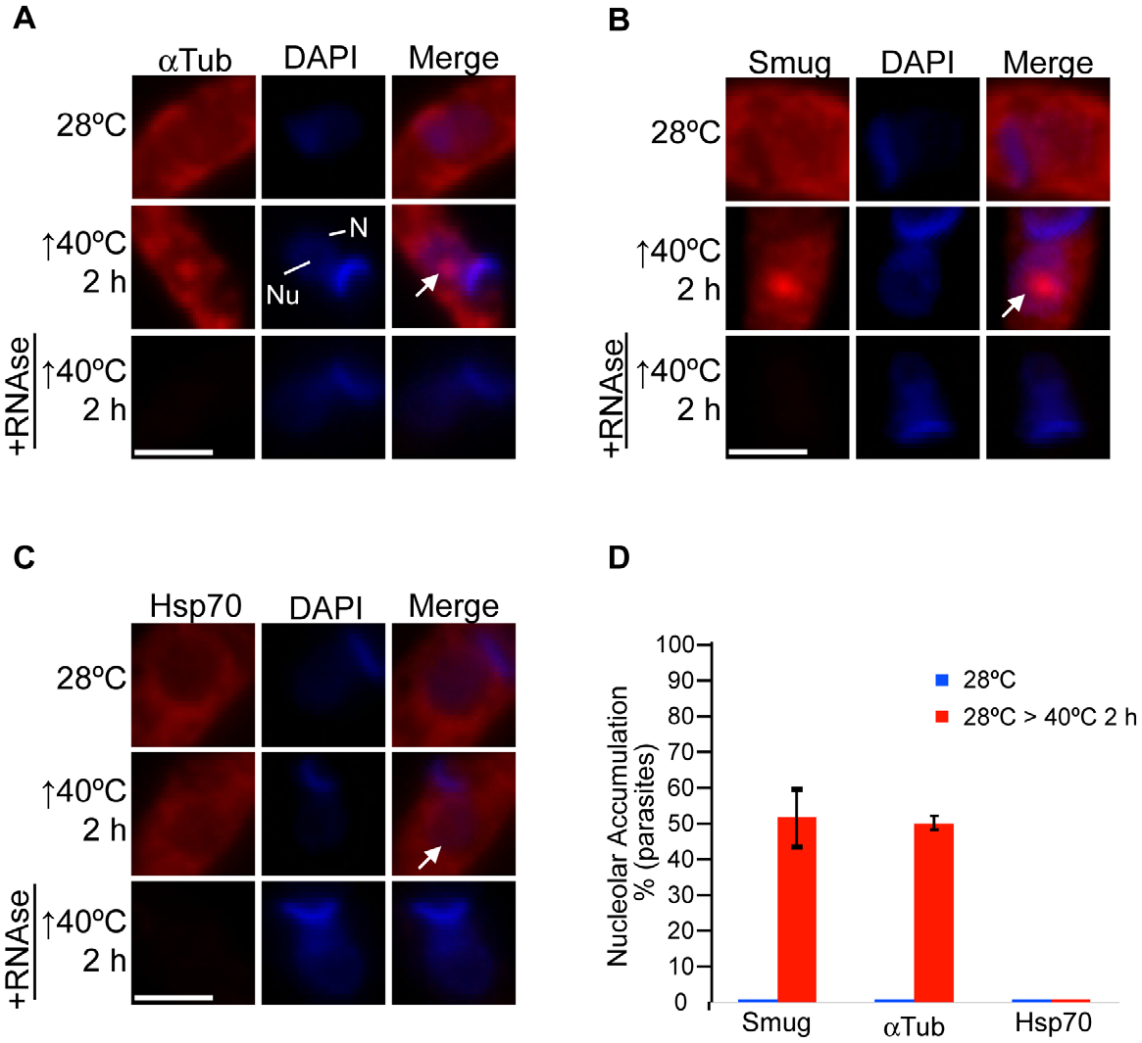
Hsp70 mRNA is able to bypass the nucleolar accumulation. FISH analysis for (A) α-Tub, (B) Smug and (C) Hsp70 mRNAs in parasites subjected to heat shock at 40°C for 2 h. In addition, cells were pre-treated with RNase A before performing FISH (bottom panels). The white arrows indicate the nucleolus. Nuclei were counterstained with DAPI (blue). Size bars represent 2 µm. Representative nuclei are shown. (D) A quantitative analysis of the experiments shown in panels (A), (B) and (C). The results are expressed as mean +/− SD from at least three independent experiments (a minimum of 100 parasites per experiment were counted).

In yeast cells, the poly(A)+ RNA is accumulated into the nucleolus in response to severe heat shock [23], [24]. Interestingly, under this condition, the export of mRNAs, such as that of Hsp70, required to mount the stress response is not inhibited [23]. To test whether a selective nucleolar accumulation of mRNAs could also take place when subjecting *T. cruzi* to severe heat shock, we evaluated the behaviour of two well-characterized mRNAs in trypanosomes: α-Tubulin (Tc00.1047053411235.9) and Hsp70 (Tc00.1047053511211.160, Tc00.1047053511211.170) [28]–[31]. It is worth mentioning that the Hsp70 mRNAs, in clear contrast with most mRNAs, are continuously translated even when parasites are subjected to severe heat shock [9], [28]. Under normal conditions, both transcripts showed mainly a cytoplasmic localization (Figure 2A and C, top panels, respectively; S2 and S3). However, after incubating parasites at 40°C for 2 h, both mRNAs showed a different sub-cellular distribution. As expected, a significant fraction of the α-Tubulin mRNA pool showed a nucleolar accumulation in 50% of the parasites (Figure 2A, middle panels, S2 and S3). On the other hand, the Hsp70 mRNA did not show a significant alteration in its localization pattern, being mainly localized throughout the cytoplasm (Figure 2C, middle panels, S2 and S3). These results suggest that in *T. cruzi*, mRNAs of proteins required to activate cellular defence mechanisms under stressed conditions are refractory to nucleolar accumulation, as it has been previously shown in yeast [23].

To further support that non-heat-shock response mRNAs are accumulated into the nucleolus under severe heat shock, we evaluated the distribution of mRNAs belonging to the Small Mucin-like family (Smug) [32], [33]. We performed the assay using a probe that could potentially hybridize with all its family members. Under normal conditions (Figure 2B, top panels, S2 and S3) Smug mRNAs were distributed throughout the cytoplasm similarly to both Hsp70 and α-Tubulin mRNAs. When parasites were subjected to heat shock, the Smug mRNAs were partially accumulated into the nucleolus in 52% of the parasites (Figure 2, middle panels, S2 and S3).

To validate that the *in situ* hybridization assays were specifically detecting the mRNAs evaluated, we performed several control experiments. The addition of an excess of the unlabelled oligonucleotide probe to the hybridization buffer significantly decreased the signal intensity for each mRNA analysed (Figure S4, S5, S6), whereas the addition of the same amount of an unlabelled oligonucleotide generated by randomizing the corresponding probe sequence did not significantly affect the intensity of the mRNA signal (Figure S4, S5, S6). In addition, we performed RT-PCR using each probe as a reverse primer in combination with a forward primer against the mini-exon sequence, which hybridizes with all mRNAs. For each mRNA evaluated, we observed only one band of the expected size, confirming the specificity of each probe by an independent approach (Figure S7). Finally, pre-treatment with RNase A before the hybridization steps almost completely abolished the nucleolar signal in heat-shocked parasites, thus demonstrating the RNA nature of the signals observed (Figure 2A–C, bottom panels).

Together, these results suggest that severe heat shock induces a selective nucleolar accumulation of mRNAs, where those mRNAs that codify for proteins not directly involved in the heat shock response are susceptible to be partially relocalized into the nucleolus; in contrast, mRNAs codifying proteins required for the heat shock response, for instance Hsp70, could bypass such nucleolar retention.

### Nucleolar Accumulation of Poly(A)+ RNA is Absent in *T. brucei*

**Figure 3:**
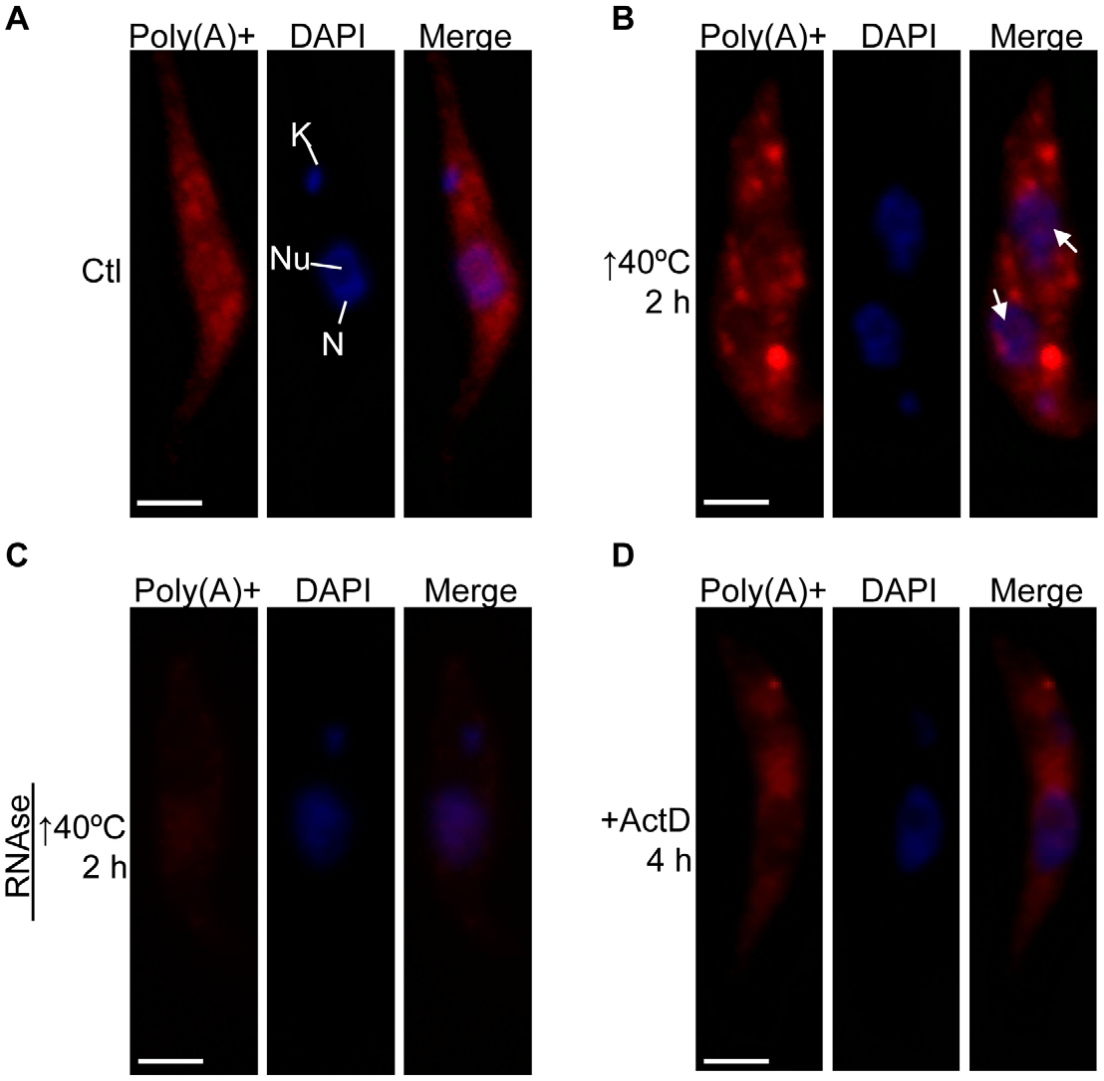
Poly(A)+ RNA in response to heat shock is located in cytoplasmic granules but not into the nucleolus in *T. brucei*. Poly(A)+ RNA subcellular localization in *T. brucei* procyclic forms (A) untreated, (B) exposed to heat shock at 40°C for 2 h, (C) pre-treated with RNase A after heat shock, and (D) incubated with ActD for 4 h. The white arrows indicate the nucleolus. Nuclei were counterstained with DAPI (blue). N: nucleus, K: kinetoplast, Nu: nucleolus. Size bars represent 2 µm. Representative parasites are shown.

Recently, the analysis of several Stress Granules (SG) protein markers (for instance TbPABP2) has shown that severe heat shock leads to SG formation in *T. brucei* [9]. Therefore, we wondered whether a fraction of the poly(A)+ RNA population could also be accumulated into the nucleolus in response to such stress. Under normal conditions (Figure 3A), the poly(A)+ RNA population was distributed throughout the cytoplasm, similarly to that observed in *T. cruzi*. Nevertheless, after subjecting *T. brucei* procyclic forms to heat shock at 40°C for 2 h, the poly(A)+ RNA distribution was notably modified, being the formation of cytoplasmic stress granules the most remarkable structures observed in almost the whole population of parasites (Figure 3B and S8). However, we did not observe nucleolar accumulation of poly(A)+ RNA under this stress condition. As previously reported by us, poly(A)+ RNA nucleolar relocalization can be induced by ActD treatment in *T. cruzi* [26]. So, we also tested this treatment in *T. brucei*. Under such condition we did not observe obvious nucleolar accumulation (compare Figure 3A and 3D); instead the poly(A)+ RNA population remained distributed throughout the cytoplasm, but in lower amounts than those observed in untreated parasites. We repeated the experiment evaluating longer times (24 h) and found only a higher cytoplasmic signal decay (not shown). These results are in agreement with the absence of nucleolar relocalization of RBPs in response to ActD treatment reported by our group in *T. brucei* [27].

Taken together, these results indicate that in *T. brucei* neither poly(A)+ RNA nor RBPs are mobilized to the nucleolus in response to stress conditions.

## Discussion

We have recently reported that several RBPs involved in the mRNA metabolism of *T. cruzi,* as well as the poly(A)+ RNA, are accumulated into the nucleolus in response to ActD treatment [26]. In this frame, we aimed to further characterize the potential role of the nucleolus in mRNA metabolism from a “physiological” perspective. Therefore, we focused our attention on severe heat shock, since this stress has been reported to induce a reduction in transcription levels in trypanosomes [9], [34], and, more importantly, because it is an environmental stress to which *T. cruzi* could be exposed during its life cycle [31]. In this work, we found that the bulk of the mRNA population is partially relocalized to the nucleolus when *T. cruzi* epimastigotes are subjected to heat shock at 40°C. In addition, we showed that this process was fully reversed when parasites were shifted to normal growth conditions (see Figure 1A and B, panels 3).

It should be mentioned that, in addition to transcription, trans-splicing was significantly reduced when exposing parasites at 40°C since we detected an accumulation of the SL RNA (Figure S9). Along this line, we have also observed a nucleolar accumulation of SL RNA (see Figure S10). In this regard, it is possible that partially processed mRNAs could also have accumulated in the nucleolus. However, we believe that the observed signals likely correspond to mature mRNAs since, as reported, transcription inhibition prevents the accumulation of incompletely processed mRNAs [35], [36]. In addition, it has been shown that such incompletely processed mRNAs could not be detected by RNA FISH, even under conditions where these mRNAs are significantly accumulated [36]. Taking this into consideration, our results showing that poly(A)+ RNA is accumulated in the nucleolus even when inducing transcription inhibition before exposing parasites to heat shock suggest that the signals observed correspond to processed mRNAs. We also showed that these mRNAs were present before subjecting parasites to heat shock (see Figure S11 IV).

**Figure 4:**
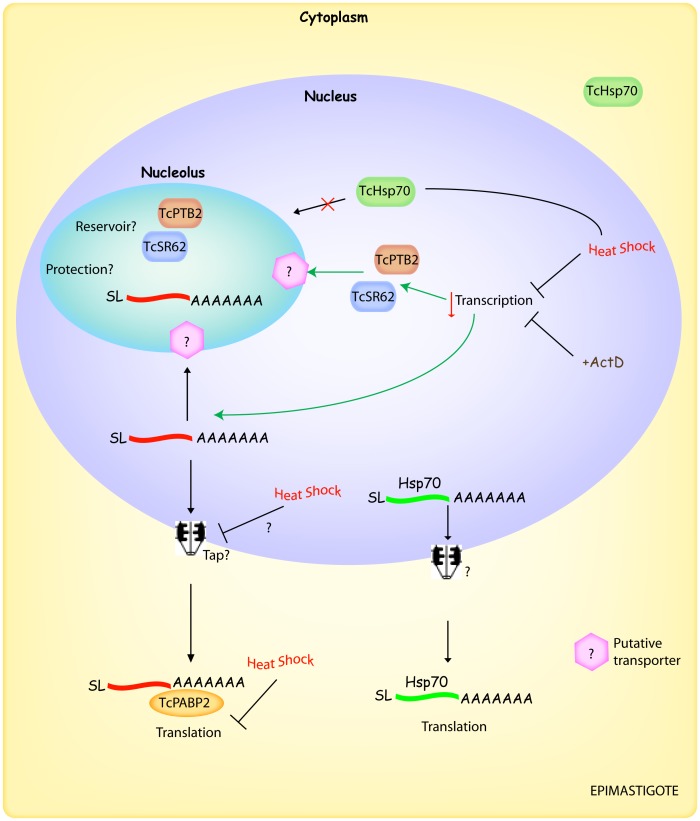
Integrative model showing the behaviour of RBPs and mRNAs in response to severe heat shock in *T. cruzi*. Under normal conditions, the mRNAs are exported to the cytoplasm to be target either to translation, storage or degradation. When epimastigotes are exposed to severe heat shock, there is a decrease in transcription which likely triggers the nucleolar accumulation of certain RBPs (for instance, TcSR62 and TcPTB2). In addition, mRNAs are relocalized into the nucleolus where they might be stored/protected until favourable environmental conditions are resumed. On the other hand, the Hsp70 mRNA could bypass such nucleolar retention, being transported to the cytoplasm where its translation can continue even under severe heat shock. Unidentified, but probable, factors/events are indicated with a question mark. See the discussion section for details. SL, spliced leader.

It has been previously reported that mRNA export in yeast is also affected by heat shock [23], [24]. In this regard, we could not initially discard, the notion that the mRNA nucleo-cytoplasmic transport might also be affected, thus resulting in the observed mRNA nucleolar accumulation (see Figure 4). However, previously reported data from experiments performed with *T. cruzi* showed that mRNA export inhibition in response either to Leptomycin B [37] or sodium arsenite [38] led to a nuclear, but no nucleolar, accumulation of mRNAs. This evidence favours the argument that the observed nucleolar accumulation of mRNAs would not be a “secondary effect” generated as a result of an mRNA export arrest.

Interestingly, the Hsp70 mRNA bypassed the nucleolar accumulation in heat-shocked parasites, suggesting that retention of mRNAs into the nucleolus might occur in a selective fashion. This observation is in agreement with previous works performed in yeast exposed to severe heat shock, showing that the bulk population of poly(A)+ RNA was accumulated into the nucleolus [23], [24]; but, the SSA4 mRNA (which codifies for Hsp70) was distributed throughout the cytoplasm under the same condition [23]. As proposed by Saavedra et al., this difference in localization is likely based on the presence of multiple signals at the sequence and/or structure level in those mRNAs that avoid such retention leading to their export, even when cells are subjected to severe heat shock conditions [23].

We next aimed to determine whether the RBPs that are mobilized to the nucleolus during stress conditions could directly participate in the nucleolar accumulation of the poly(A)+ RNA population. Since *T. cruzi* does not have a full RNAi machinery [39], [40], we attempted an antisense knockdown strategy [41] to inhibit the expression of different RBPs. This strategy did not work, and we were thus not able to dissect the role of the RBPs in the nucleolar accumulation of poly(A)+ RNA.

We have previously reported that the mechanism driving the nucleolar relocalization of RBPs, induced by transcription inhibition, is not functional in *T. brucei* [27]. Here, we extended these findings by showing that the nucleolar accumulation of poly(A)+ RNA is also absent in this parasite. So, both results strongly indicate that, unlike that observed in *T. cruzi*, the *T. brucei* nucleolus does not play a role in mRNA metabolism during stress conditions, and also suggest that this role has been differently conserved in the trypanosomatid lineage. Strikingly, it seems that trypanosomatids have developed different responses to deal with the same stress condition. For instance, confronted to severe heat shock, *T. brucei* responds with the formation of cytoplasmic SG (see Figure S8) [9], while *T. cruzi* accumulates poly(A)+ and certain RBPs [26] in its nucleolus, without apparent SG formation. It should be highlighted that, to our knowledge, the nucleolar accumulation of mRNAs in response to severe heat shock has been observed only in yeast and *T. cruzi* (this work). Taking this information into account, an interesting question is why mRNAs need to be stored in the nucleolus in response to heat shock in *T. cruzi*. It has been previously shown that cytoplasmic poly(A)+ RNA granule formation induced by starvation persisted after ActD treatment in trypanosomes, thus indicating that the mRNA is stabilized in such structures [7]. On the other hand, it has been reported in mammals that ActD treatment leads to the loss of P bodies favouring the argument that transcripts in those structures are degraded if there is not enough supply of mRNAs to support them [42]. Based on these observations, if the *T. cruzi* nucleolus were a focus of mRNA protection, then we would expect that the nucleolar mRNA in heat-shocked parasites might remain even after treating with ActD. Preliminary experiments showed that the nucleolar poly(A)+ RNA signal still persisted under such conditions (Figure S11 V). Moreover, we have previously demonstrated that long-term incubation with ActD results in cytoplasmic poly(A)+ RNA decay, but also nucleolar mRNA accumulation, even after 24 h of ActD treatment [26]. Together, these results suggest a potential role of the nucleolus as a protective structure for mRNAs.

Together, our results raise the interesting possibility that the *T. cruzi* nucleolus plays an important role in the response to environmental stress conditions, such as severe heat shock (outlined in Figure 4). Under this condition, the nucleolus may behave as a storage/protection structure for factors involved in gene expression, such as RBPs and most mRNAs, until favourable conditions are restored. However, factors (for instance Hsp70) and mRNAs codifying proteins required to overcome the heat shock stress bypass the nucleolar retention.

These results also support the idea that the additional functions of the nucleolus might be already present in ancestral organisms such as trypanosomes, arguing in favour of the fact that this potential alternative way of post-transcriptional gene regulation could have been acquired early in the evolution of the eukaryotic cell.

## Materials and Methods

### Parasites

*T. cruzi* epimastigotes (CL strain) were cultured in BHT 10% medium (brain heart infusion, 0.3% tryptose, 0.002% bovine hemin) supplemented with 10% heat-inactivated fetal calf serum, streptomycin 0.1 mg/ml and penicillin 100 U/ml at 28°C. *T. brucei* procyclic (Lister 427 strain) form cells were cultured in SDM79 medium. Parasite cultures were taken in a late logarithmic growth phase at a cell density of 2.5−3.5×10^7^/ml parasites for *T. cruzi* and of 0.5×10^7^/ml parasites for *T. brucei*.

### Treatments

For the heat shock experiments, parasites were incubated in a water bath at 40°C. Transcription inhibition was induced incubating parasites with ActD 50 µg/ml.

### RNA Preparation

Total RNA was isolated using TRIzol reagent (Invitrogen) and residual genomic DNA was removed by DNase treatment using RNase-free DNase I (Ambion). Both procedures were performed according to the manufacturer’s instructions.

### Immunofluorescence

Trypanosomes were centrifuged from log phase cultures for 2 minutes at 2000 g, washed in PBS twice, allowed to settle on poly-L-lysine-coated slides and fixed in paraformaldehyde (PFA) 4% in PBS at room temperature (RT) for 10 minutes. After two brief washes in PBS at RT, fixed cells were incubated at RT with 25 mM NH_4_Cl in PBS for 10 minutes. Cells were washed twice with PBS, permeabilized and blocked with 0.5% saponin, 1% Bovine serum albumin, 2% goat normal serum in PBS for 60 minutes at RT. After blocking, cells were first incubated with the primary antibody (diluted in 0.1% saponin and 1% BSA in PBS) for 60 minutes and then washed 3 times with PBS. Afterwards, slides were incubated with secondary antibodies (diluted in 0.1% saponin and 1% BSA in PBS) for 60 minutes, washed three times with PBS and once in Milli-Q water. The primary antibody was polyclonal anti-TcPABP2 (1∶1000). The secondary goat anti-rabbit antibody AlexaFluor 488 (Molecular Probes) was used at 1∶1000 dilutions.

### Fluorescence *in situ* Hybridization (FISH)

For detection of total poly(A)+ RNA by FISH, parasites were harvested, allowed to adhere to poly-L-lysine-coated microscope slides, fixed with 4% paraformaldehyde in PBS at RT for 10 minutes, followed by a 10-min incubation with 25 mM NH_4_Cl. Fixed parasites were permeabilized and blocked for 1 h in 0.5% saponin (Sigma), 2% BSA (Blocking Buffer), followed by 2-h prehybridization at RT in 2% BSA, 5X Denhardt, 4X SSC, 5% dextran sulphate, 35% deionized formamide (Sigma), 0.5 µg/µl yeast tRNA (Sigma) and 10 U/ml RNasin (Promega) (Hybridization Solution). Hybridizations were performed overnight at 28°C in a humid chamber either in the presence of 1 ng/µl Cy3-conjugated oligo-(dT)30 or Cy3-conjugated oligo-(dA)30 in Hybridization Solution. Slides were washed twice in 4X SSC at RT. Slides were mounted in 1 µg/ml DAPI prepared in Fluorsave (Calbiochem). RNase A pretreatment was performed at 37°C for 30 minutes before hybridization.

The following probes were also used:

- Mini-exon: *caatatagtacagaaactgtatcaataatagGgtt
- α-Tub (Tc00.1047053411235.9):
  - ○ *CGACGAGTTAAATCATAAATTGCTT
  - ○Random α-Tub: CCGATTGATGATCATAATAAATGTC
- Smug (Tc00.1047053504539.30, Tc00.1047053504539.20, Tc00.1047053504539.10):
  - ○ *CTCAAACACAGCAGCATCGT
  - ○ Random Smug: CATCCACATAAGGGTCCAAC
- Hsp70 (Tc00.1047053511211.160, Tc00.1047053511211.170):
  - ○ *CAATCTCCTTCATCTTTGACAGGAC
  - ○Random Hsp70: CATCGCCCAGTGTTATTTCCAATCA

The asterisks indicate the position of Cy3 in each probe.

## Supporting Information

Figure S1 **Subcellular localization of the oligo(dT) and mini-exon probes under normal and severe heat shock conditions.** Representative field sections showing the localization of oligo(dT) and mini-exon probes in untreated parasites and parasites subjected to heat shock at 40°C for 2 h. Both probes are shown in red. Nuclei were counterstained with DAPI (blue). Size bars represent 10 µm.
(TIF)

Figure S2 **Subcellular localization of α-Tub, Smug and Hsp70 mRNAs under normal and severe heat shock conditions.** Representative field sections showing the localization of the corresponding mRNAs are shown in untreated parasites and parasites subjected to heat shock at 40°C for 2 h. mRNAs are shown in red. Nuclei were counterstained with DAPI (blue). Size bars represent 10 µm.
(TIF)

Figure S3 **Effects of severe heat shock on the localization of poly(A)+, mini-exon, α-Tub, Smug and Hsp70 RNAs showing whole parasites.** FISH images of the corresponding RNAs in heat shock-treated and untreated epimastigotes. Nuclei were counterstained with DAPI (blue). The third column on the right is an overlap of each RNA and DNA staining. The white arrows indicate the nucleolus. Size bars represent 2 µm. Representative parasites are shown.
(TIF)

Figure S4 **Specificity analysis of the α-Tub probe by competition assays.** Competitions were performed by adding either a molar excess of the corresponding randomized unlabeled probe (middle panels) or the corresponding unlabeled probe (bottom panels) in the Hybridization Solution containing the corresponding Cy3 labelled probe. Nuclei were counterstained with DAPI (blue). Size bars represent 10 µm. Representative fields are shown.
(TIF)

Figure S5 **Specificity analysis of the Smug probe by competition assays.** Competitions were performed by adding either a molar excess of the corresponding randomized unlabelled probe (middle panels) or the corresponding unlabelled probe (bottom panels) in the Hybridization Solution containing the corresponding Cy3 labelled probe. Nuclei were counterstained with DAPI (blue). Size bars represent 10 µm. Representative fields are shown.
(TIF)

Figure S6 **Specificity analysis of the Hsp70 probe by competition assays.** Competitions were performed by adding either a molar excess of the corresponding randomized unlabelled probe (middle panels) or the corresponding unlabelled probe (bottom panels) in the Hybridization Solution containing the corresponding Cy3 labelled probe. Nuclei were counterstained with DAPI (blue). Size bars represent 10 µm. Representative fields are shown.
(TIF)

Figure S7 **Specificity analysis of the α-Tub, Smug and Hsp70 probes by RT- PCR.** RT-PCR using each probe as a reverse primer in combination with a forward primer (AACGCTATTATTGATACAGTTTCTGT) against the mini-exon sequence, in untreated parasites and parasites exposed to heat shock at 40°C for 2 h.
(TIF)

Figure S8 **Colocalization of poly(A)+ RNA granules and TbPABP2 in response to heat shock in** ***T. brucei*** **.** Immunofluorescence against TbPABP2 (green), a stress granule marker, coupled to FISH using a Cy3-labelled oligo(dT)30 probe (red) in parasites that were untreated or exposed to heat shock at 40°C for 2 h. Representative parasites are shown. Size bar represents 2 µm.
(TIF)

Figure S9 **SL RNA accumulation in response to heat shock.** (A) Semi-quantitative RT-PCR against the SL RNA in parasites that were untreated or exposed to heat shock at 40°C during 2 h was performed taking samples at the following PCR cycles: 25, 28 and 30. SL primers (Fw: TGATACAGTTTCTGTACTATATTGGTACG; Rv: TGGACCACGGTCAAAAGAA). U3 snoRNA was used as a loading control (U3 primers Fw: CCGTACTCTGAACAGAATCG; Rv: CCAGCAACCTTCATCATCAG). cDNA was synthesized by using random hexamers. PCR cycle conditions: 94°C 30″, 50°C 30″, 72°C 30″. A quantitative analysis of the experiments is shown in panel (B). The results are expressed as mean +/− SD from three independent experiments.
(TIF)

Figure S10 **SL RNA nucleolar accumulation in response to heat shock.** RNA FISH analysis for SL RNA (Probe: HEX*AAAGGGTTCGTGGACCCC) in untreated parasites and parasites incubated at 40°C for 2 h. Nuclei were counterstained with DAPI (blue). Representative parasites are shown. Size bar represents 2 µm.
(TIF)

Figure S11 **Nucleolar accumulation of poly(A)+ RNA in response to heat shock is not prevented either before or after ActD treatment.** Poly(A)+ RNA (red) was analyzed in epimastigotes pre-treated with ActD for 1 h and then exposed to heat shock at 40°C during 2 h; or exposed to heat shock at 40°C during 1 h and then treated with ActD for 2 h. Treatments alone are shown as controls. Nuclei were counterstained with DAPI (blue). Representative parasites are shown. Size bar represents 2 µm.
(TIF)
