# Supplementary figures and images for: Severe Heat Shock Induces Nucleolar Accumulation of mRNAs in Trypanosoma cruzi
Source: PLoS One. 2012 Aug 27;7(8):e43715. doi: 10.1371/journal.pone.0043715 (PMC3428281; doi:10.1371/journal.pone.0043715)

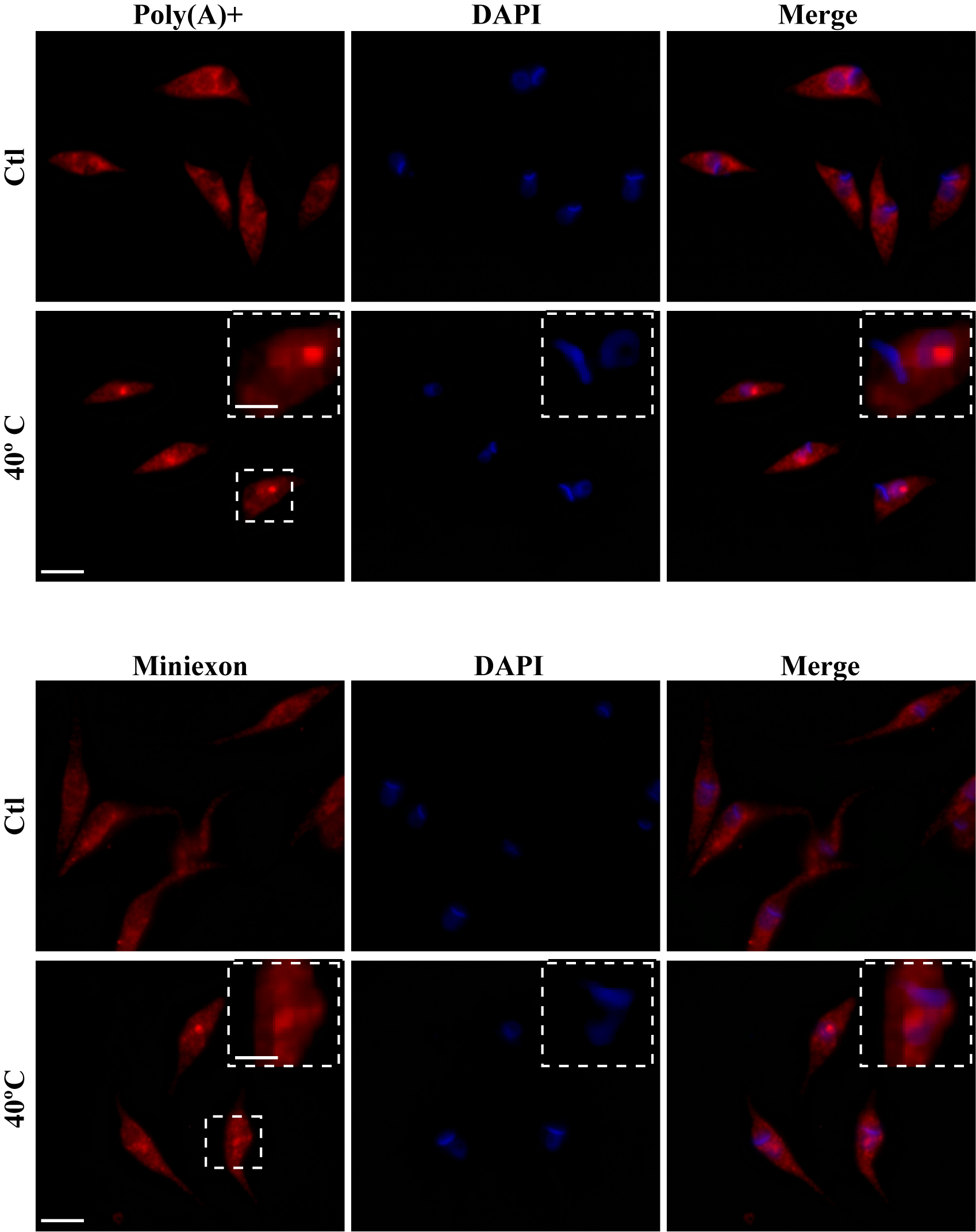

Supplement: Figure S1 — Subcellular localization of the oligo(dT) and mini-exon probes under normal and severe heat shock conditions. Representative field sections showing the localization of oligo(dT) and mini-exon probes in untreated parasites and parasites subjected to heat shock at 40°C for 2 h. Both probes are shown in red. Nuclei were counterstained with DAPI (blue). Size bars represent 10 µm. (TIF) [file pone.0043715.s001.tif]

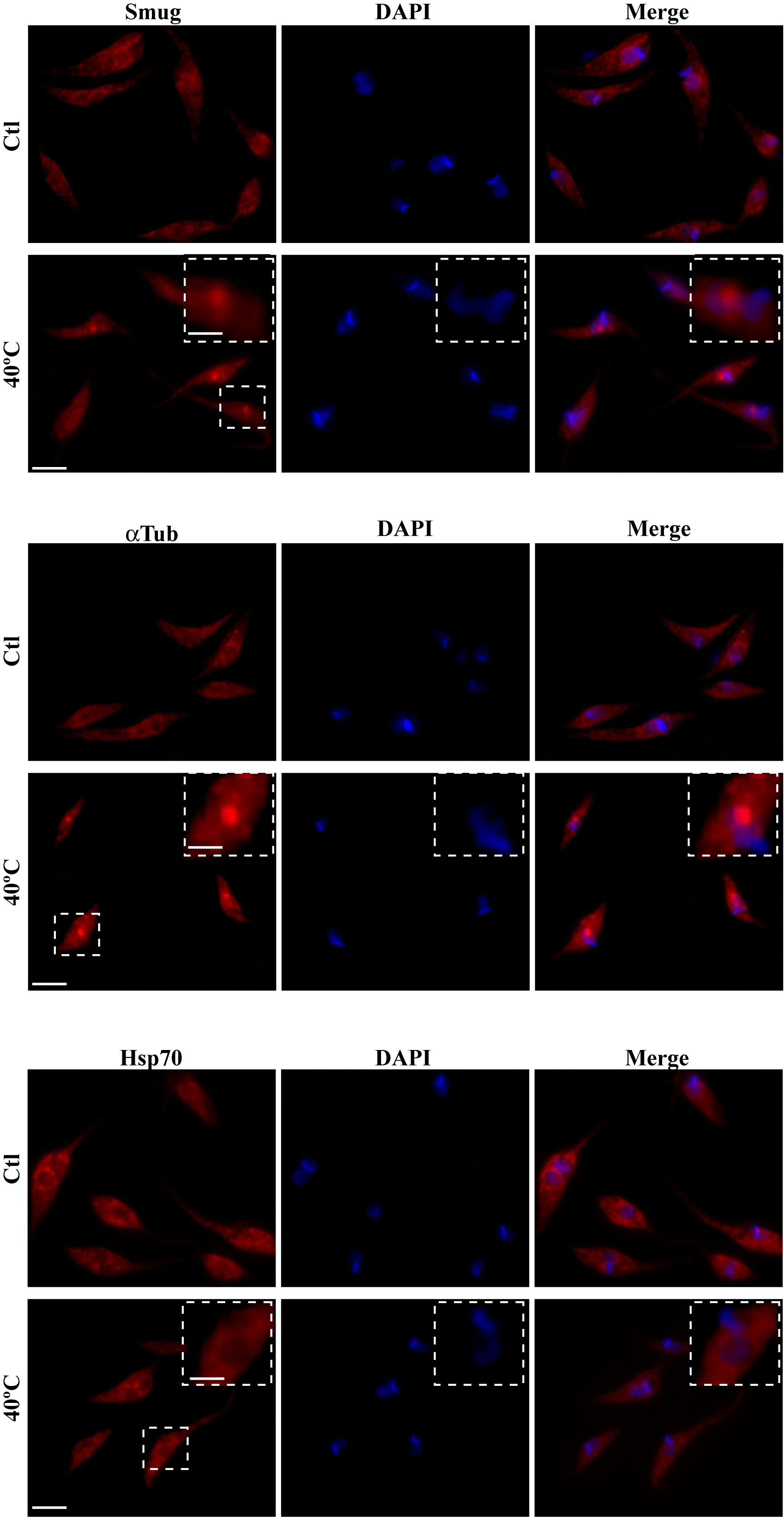

Supplement: Figure S2 — Subcellular localization of α-Tub, Smug and Hsp70 mRNAs under normal and severe heat shock conditions. Representative field sections showing the localization of the corresponding mRNAs are shown in untreated parasites and parasites subjected to heat shock at 40°C for 2 h. mRNAs are shown in red. Nuclei were counterstained with DAPI (blue). Size bars represent 10 µm. (TIF) [file pone.0043715.s002.tif]

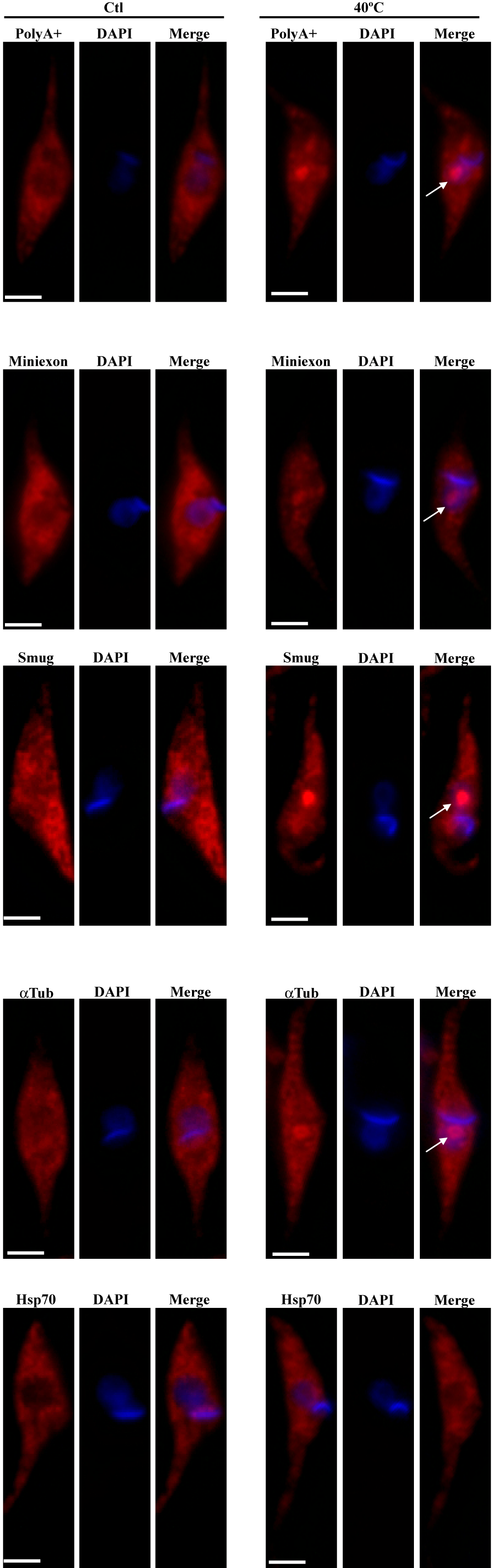

Supplement: Figure S3 — Effects of severe heat shock on the localization of poly(A)+, mini-exon, α-Tub, Smug and Hsp70 RNAs showing whole parasites. FISH images of the corresponding RNAs in heat shock-treated and untreated epimastigotes. Nuclei were counterstained with DAPI (blue). The third column on the right is an overlap of each RNA and DNA staining. The white arrows indicate the nucleolus. Size bars represent 2 µm. Representative parasites are shown. (TIF) [file pone.0043715.s003.tif]

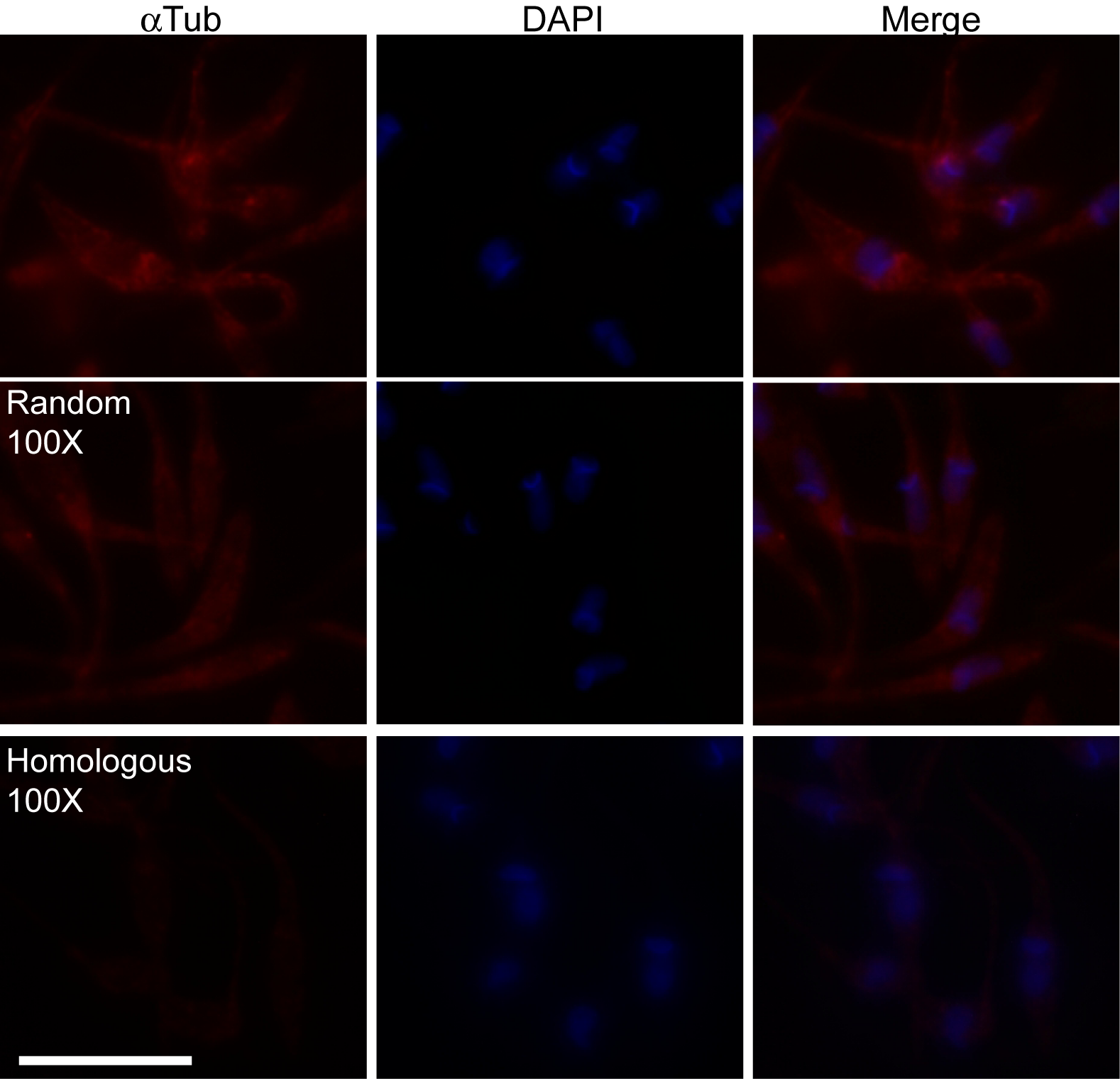

Supplement: Figure S4 — Specificity analysis of the α-Tub probe by competition assays. Competitions were performed by adding either a molar excess of the corresponding randomized unlabeled probe (middle panels) or the corresponding unlabeled probe (bottom panels) in the Hybridization Solution containing the corresponding Cy3 labelled probe. Nuclei were counterstained with DAPI (blue). Size bars represent 10 µm. Representative fields are shown. (TIF) [file pone.0043715.s004.tif]

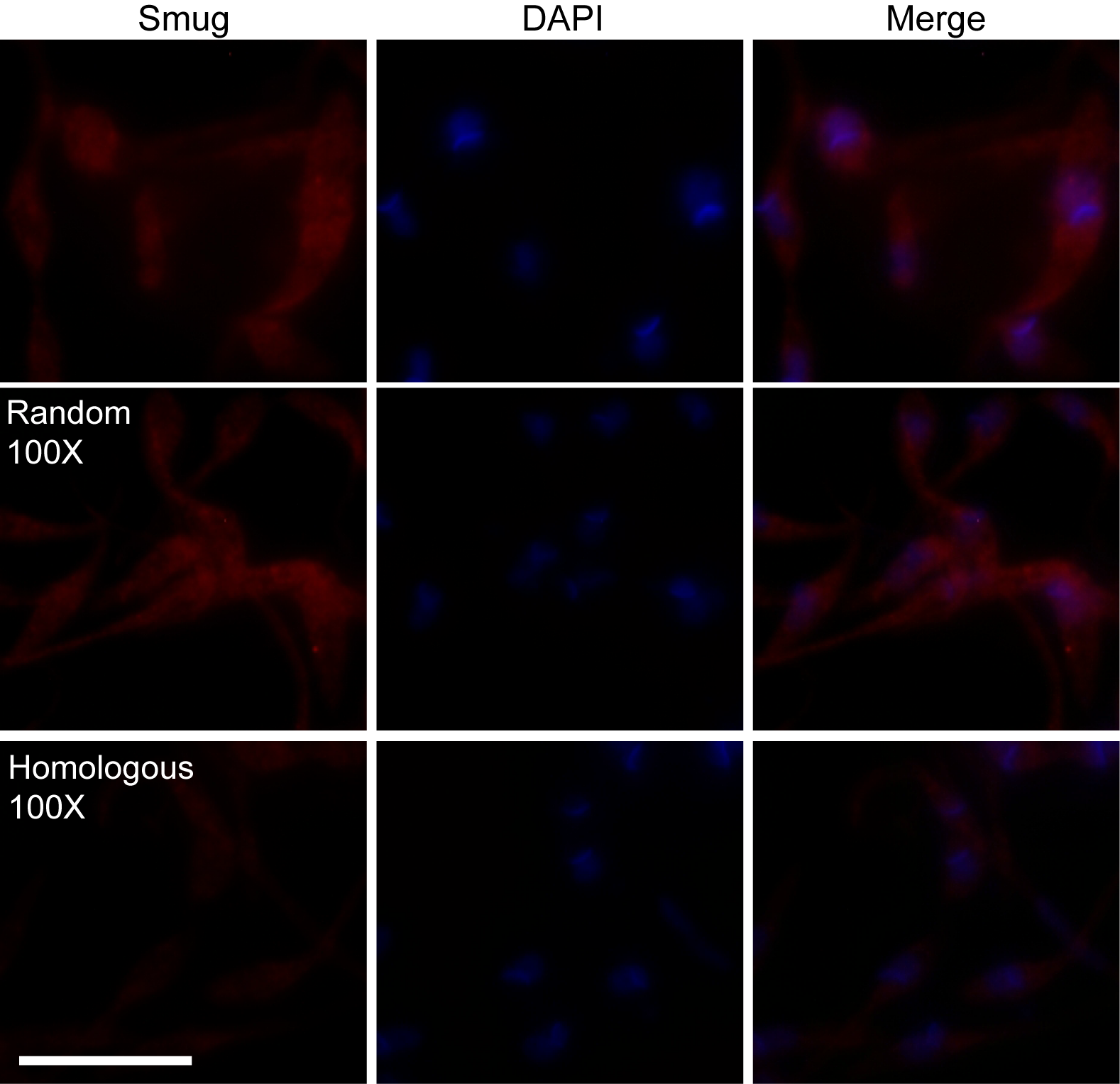

Supplement: Figure S5 — Specificity analysis of the Smug probe by competition assays. Competitions were performed by adding either a molar excess of the corresponding randomized unlabelled probe (middle panels) or the corresponding unlabelled probe (bottom panels) in the Hybridization Solution containing the corresponding Cy3 labelled probe. Nuclei were counterstained with DAPI (blue). Size bars represent 10 µm. Representative fields are shown. (TIF) [file pone.0043715.s005.tif]

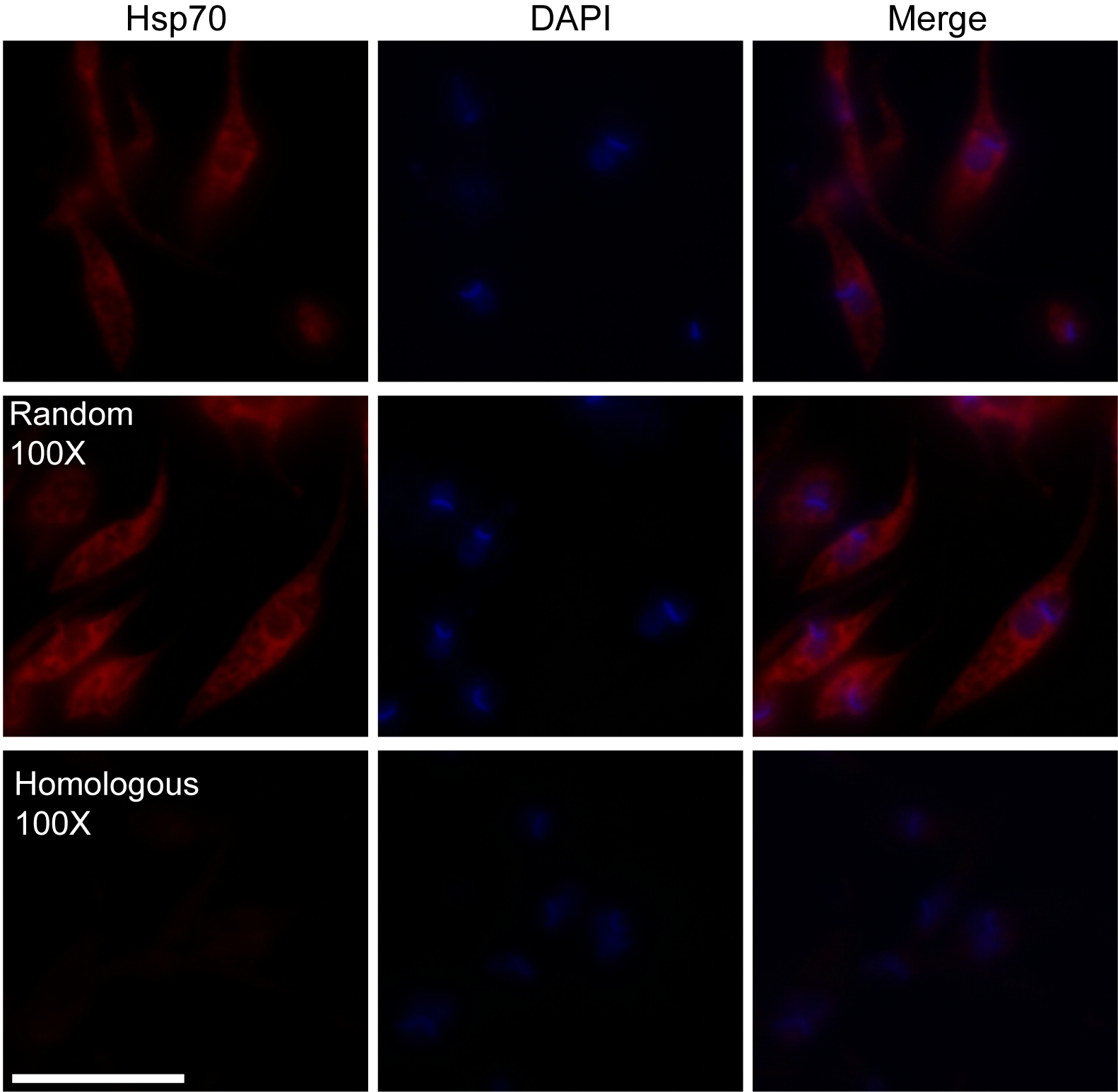

Supplement: Figure S6 — Specificity analysis of the Hsp70 probe by competition assays. Competitions were performed by adding either a molar excess of the corresponding randomized unlabelled probe (middle panels) or the corresponding unlabelled probe (bottom panels) in the Hybridization Solution containing the corresponding Cy3 labelled probe. Nuclei were counterstained with DAPI (blue). Size bars represent 10 µm. Representative fields are shown. (TIF) [file pone.0043715.s006.tif]

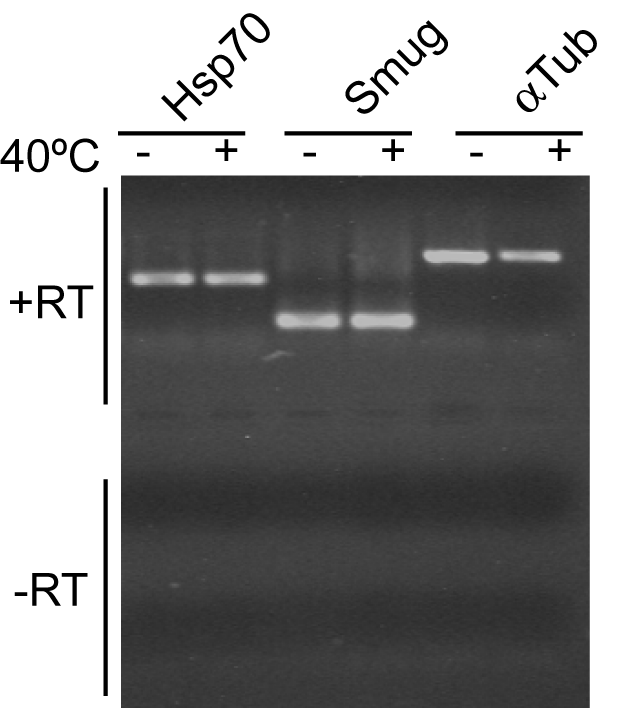

Supplement: Figure S7 — Specificity analysis of the α-Tub, Smug and Hsp70 probes by RT- PCR. RT-PCR using each probe as a reverse primer in combination with a forward primer (AACGCTATTATTGATACAGTTTCTGT) against the mini-exon sequence, in untreated parasites and parasites exposed to heat shock at 40°C for 2 h. (TIF) [file pone.0043715.s007.tif]

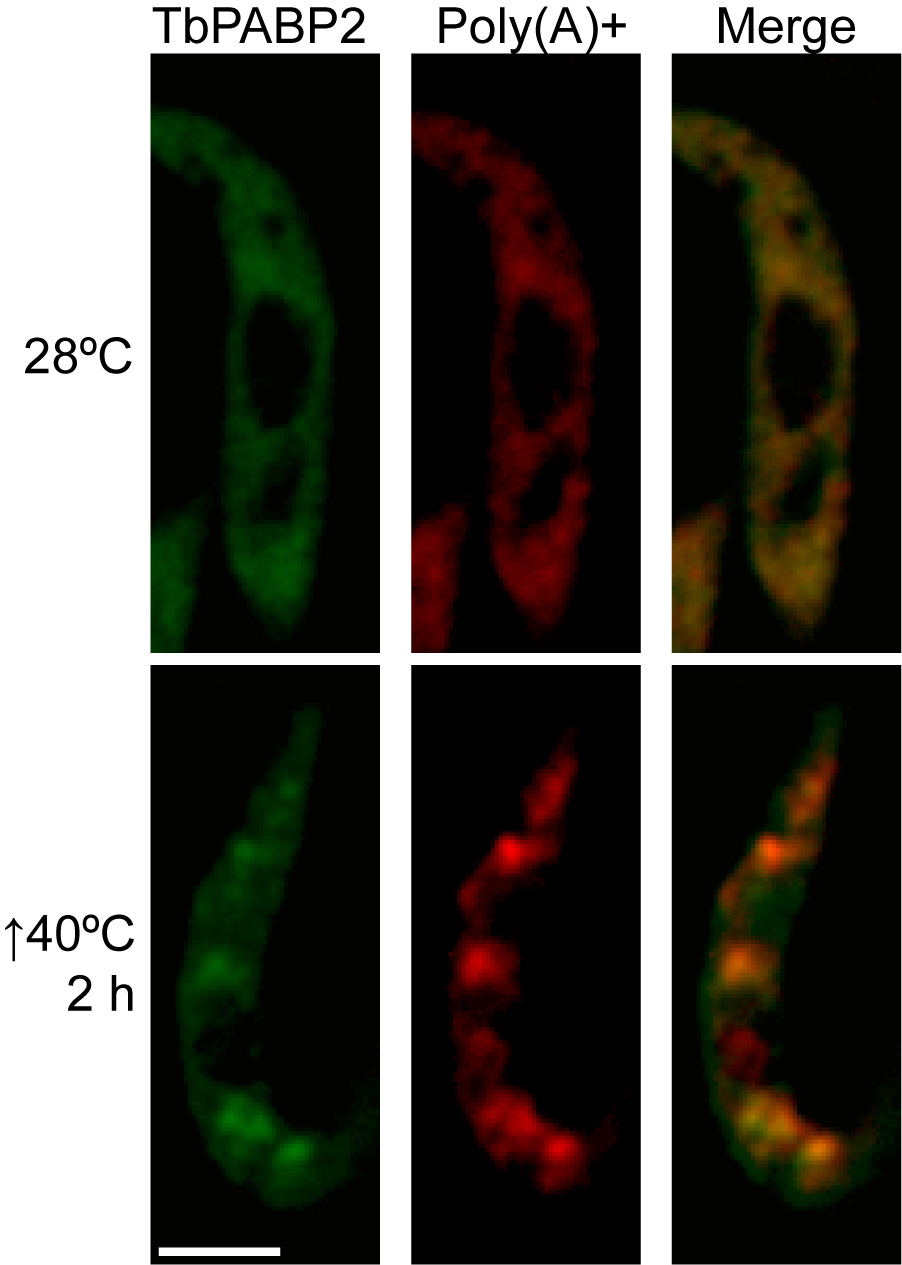

Supplement: Figure S8 — Colocalization of poly(A)+ RNA granules and TbPABP2 in response to heat shock in T. brucei . Immunofluorescence against TbPABP2 (green), a stress granule marker, coupled to FISH using a Cy3-labelled oligo(dT)30 probe (red) in parasites that were untreated or exposed to heat shock at 40°C for 2 h. Representative parasites are shown. Size bar represents 2 µm. (TIF) [file pone.0043715.s008.tif]

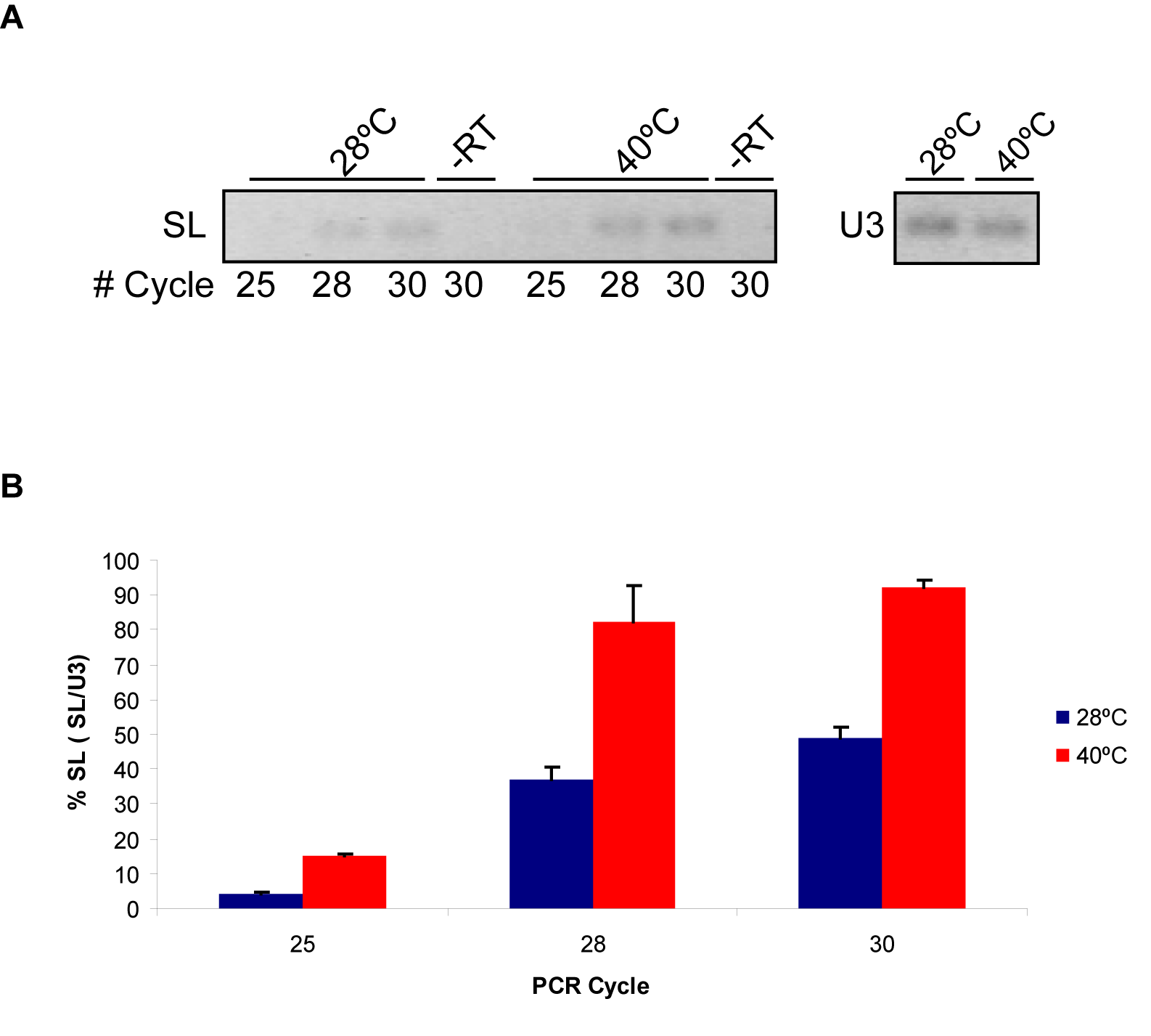

Supplement: Figure S9 — SL RNA accumulation in response to heat shock. (A) Semi-quantitative RT-PCR against the SL RNA in parasites that were untreated or exposed to heat shock at 40°C during 2 h was performed taking samples at the following PCR cycles: 25, 28 and 30. SL primers (Fw: TGATACAGTTTCTGTACTATATTGGTACG; Rv: TGGACCACGGTCAAAAGAA). U3 snoRNA was used as a loading control (U3 primers Fw: CCGTACTCTGAACAGAATCG; Rv: CCAGCAACCTTCATCATCAG). cDNA was synthesized by using random hexamers. PCR cycle conditions: 94°C 30″, 50°C 30″, 72°C 30″. A quantitative analysis of the experiments is shown in panel (B). The results are expressed as mean +/− SD from three independent experiments. (TIF) [file pone.0043715.s009.tif]

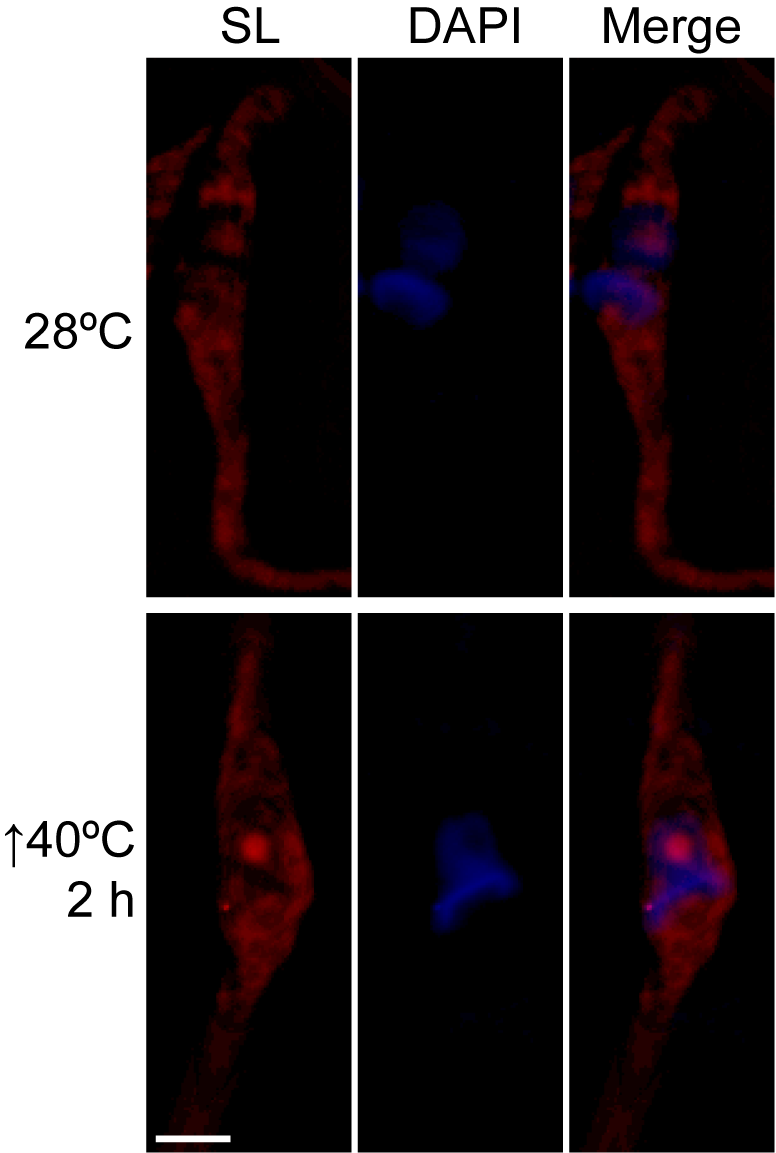

Supplement: Figure S10 — SL RNA nucleolar accumulation in response to heat shock. RNA FISH analysis for SL RNA (Probe: HEX*AAAGGGTTCGTGGACCCC) in untreated parasites and parasites incubated at 40°C for 2 h. Nuclei were counterstained with DAPI (blue). Representative parasites are shown. Size bar represents 2 µm. (TIF) [file pone.0043715.s010.tif]

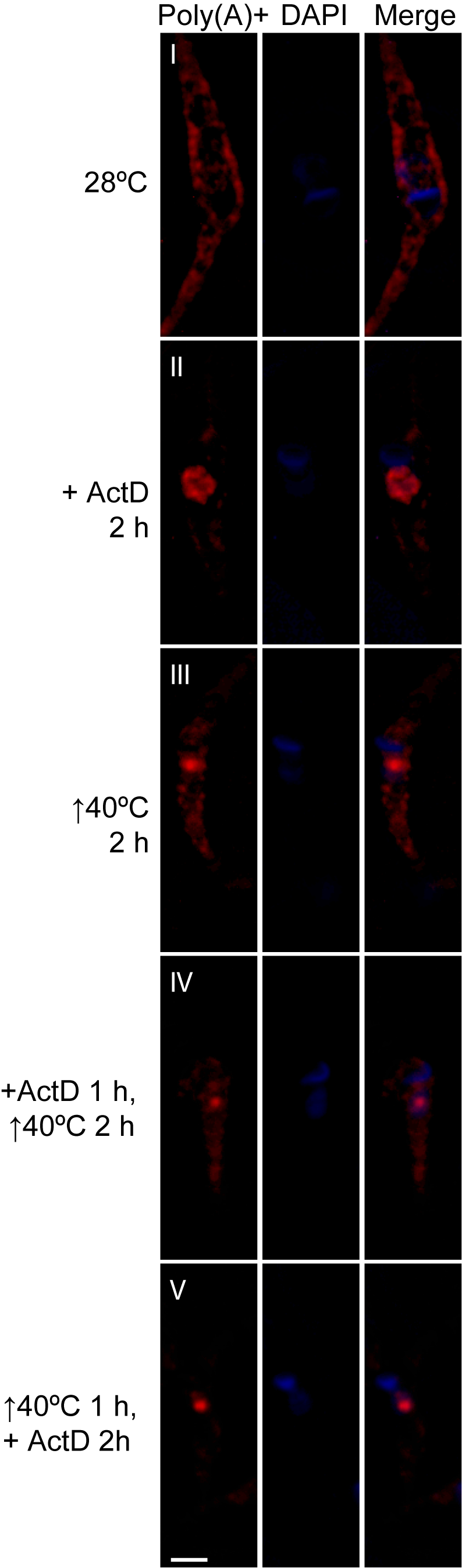

Supplement: Figure S11 — Nucleolar accumulation of poly(A)+ RNA in response to heat shock is not prevented either before or after ActD treatment. Poly(A)+ RNA (red) was analyzed in epimastigotes pre-treated with ActD for 1 h and then exposed to heat shock at 40°C during 2 h; or exposed to heat shock at 40°C during 1 h and then treated with ActD for 2 h. Treatments alone are shown as controls. Nuclei were counterstained with DAPI (blue). Representative parasites are shown. Size bar represents 2 µm. (TIF) [file pone.0043715.s011.tif]
